# Combining magnetic resonance imaging with a multi-ancestry polygenic risk score to improve identification of clinically significant prostate cancer

**DOI:** 10.1093/jncics/pkae014

**Published:** 2024-03-01

**Authors:** Anna Plym, Ikenna Madueke, Sachin Naik, Mark Isabelle, David V Conti, Christopher A Haiman, Kathryn L Penney, Lorelei A Mucci, Rhamin Khorasani, Adam S Kibel

**Affiliations:** Department of Urology, Brigham and Women’s Hospital, Harvard Medical School, Boston, MA, USA; Department of Epidemiology, Harvard T.H. Chan School of Public Health, Boston, MA, USA; Department of Medical Epidemiology and Biostatistics, Karolinska Institutet, Stockholm, Sweden; Department of Urology, Brigham and Women’s Hospital, Harvard Medical School, Boston, MA, USA; Center for Evidence-Based Imaging, Department of Radiology, Brigham and Women's Hospital, Harvard Medical School, Boston, MA, USA; Center for Evidence-Based Imaging, Department of Radiology, Brigham and Women's Hospital, Harvard Medical School, Boston, MA, USA; Center for Genetic Epidemiology, Department of Population and Public Health Sciences, Keck School of Medicine, University of Southern California, Los Angeles, CA, USA; Center for Genetic Epidemiology, Department of Population and Public Health Sciences, Keck School of Medicine, University of Southern California, Los Angeles, CA, USA; Department of Epidemiology, Harvard T.H. Chan School of Public Health, Boston, MA, USA; Channing Division of Network Medicine, Department of Medicine, Brigham and Women's Hospital and Harvard Medical School, Boston, MA, USA; Department of Epidemiology, Harvard T.H. Chan School of Public Health, Boston, MA, USA; Center for Evidence-Based Imaging, Department of Radiology, Brigham and Women's Hospital, Harvard Medical School, Boston, MA, USA; Department of Urology, Brigham and Women’s Hospital, Harvard Medical School, Boston, MA, USA

## Abstract

Multi-parametric magnetic resonance imaging (mpMRI) has emerged as an important tool for identifying clinically significant prostate cancer. We examined if the addition of a 400-variant multi-ancestry polygenic risk score (PRS) to mpMRI has the potential to improve identification. Based on data from 24 617 men from the Mass General Brigham Biobank, we identified 1243 men who underwent mpMRI. Men in the top PRS quartile were more likely to have clinically significant prostate cancer (47.1% vs 28.6% in the bottom PRS quartile, adjusted relative proportion 1.72 [95% CI = 1.35 to 2.19]). Both among men with a positive and a negative mpMRI, men in the top PRS quartile had the highest frequency of clinically significant cancer. In a constructed scenario for selecting men to undergo biopsy, use of the PRS lowered the frequency of missed clinically significant cancers from 9.1% to 5.9%. Our study provides initial support for using the PRS to improve identification of potentially lethal prostate cancer.

Multi-parametric magnetic resonance imaging (mpMRI) aids in targeting prostate lesions for biopsy and has emerged as an important tool for identifying clinically significant prostate cancer, defined as grade group 2 or higher (Gleason score 7 or higher) cancer (1-3). Based on the Prostate Imaging–Reporting and Data System (PI-RADS) v. 2.1, mpMRI has a sensitivity of 87% and a specificity of 74% to detect clinically significant cancer (4), and the proportion of clinically significant cancer increases from 0% for PI-RADS score 1 to 80% for PI-RADS score 5 (5).

While mpMRI is predominantly used in men with elevated prostate-specific antigen, men with a high genetic predisposition may be another target population. Prostate cancer is the most heritable cancer, and 451 germline risk variants for prostate cancer have been identified to date (6). Combined into a polygenic risk score (PRS), these variants have been shown to robustly stratify men according to prostate cancer risk. A criticism of the PRS has been that it cannot distinguish between prostate cancers of low and high risk of metastatic progression, ie, the PRS has, at least historically, been equally strongly associated with nonaggressive and aggressive disease (7). For a new 400-variant multi-ancestry PRS that excludes 51 variants linked to prostate-specific antigen levels (potentially influencing the ability to detect disease), a consistently stronger association with aggressive disease vs nonaggressive disease has been demonstrated (6). However, the PRS still predicts cancers without aggressive features to a large degree, and the PRS alone is not sufficient for determining which men harbor aggressive disease.

The aim of this study was to examine if the addition of a PRS to mpMRI has the potential to improve identification of clinically significant prostate cancer. From a source population of 24 617 genotyped men aged 40 years or above but without any other restriction in the Mass General Brigham Biobank, we identified 1243 men who underwent a prostate mpMRI between January 2015 and March 2021 (Table 1). Men who only had an mpMRI 1 year or later after being diagnosed with prostate cancer or after receiving treatment for prostate cancer were excluded (*n *=* *222). Clinical data were extracted from electronic health records as described in the Supplementary Material (available online). All participants provided written informed consent, and the project has been approved by the Mass General Brigham Institutional Review Board.

**Table 1.** Characteristics and biopsy results for genotyped men in the Mass General Brigham Biobank with an indication for and who underwent mpMRI between 2015 and 2021

Characteristics	Bottom PRS (0%-25%)^a^	Middle PRS (25%-75%)^a^	Top PRS (75%-100%)^a^	All men
No. of men	203	645	395	1243
Age at mpMRI, years, median (IQR)	66.0 (60.6-71.7)	66.9 (61.4-71.6)	64.6 (59.4-69.6)	66.0 (60.6-71.0)
Prostate-specific antigen, ng/mL, median (IQR)^b^	5.8 (4.2-8.4)	5.8 (4.4-8.2)	5.9 (4.5-7.7)	5.8 (4.4-8.1)
PRS, median (range)	25.0 (23.8-25.3)	25.8 (25.3-26.2)	26.6 (26.2-28.8)	25.9 (23.8-28.8)
Race, n (%)^c^				
Asian	6 (3.0)	8 (1.2)	3 (0.8)	17 (1.4)
Black	1 (0.5)	8 (1.2)	34 (8.6)	43 (3.5)
White	185 (91.1)	599 (92.9)	342 (86.6)	1126 (90.6)
Other^d^	3 (1.5)	12 (1.9)	11 (2.8)	26 (2.1)
Missing	8 (3.9)	18 (2.8)	5 (1.3)	31 (2.5)
PI-RADS score, n (%)				
≤2	93 (45.8)	290 (45.0)	135 (34.2)	518 (41.7)
3	21 (10.3)	61 (9.5)	33 (8.4)	115 (9.3)
4-5	89 (43.8)	294 (45.6)	227 (57.5)	610 (49.1)
Biopsy results, n (%)				
Grade group 1 (Gleason score 6)	20 (9.9)	111 (17.2)	89 (22.5)	220 (17.7)
Grade group 2 (Gleason score 3 + 4)	19 (9.4)	95 (14.7)	91 (23.0)	205 (16.5)
Grade group 3 (Gleason score 4 + 3)	13 (6.4)	51 (7.9)	48 (12.2)	112 (9.0)
Grade group 4 (Gleason score 8)	12 (5.9)	44 (6.8)	28 (7.1)	84 (6.8)
Grade group 5 (Gleason score 9-10)	14 (6.9)	38 (5.9)	19 (4.8)	71 (5.7)
Negative biopsy	46 (22.7)	89 (13.8)	37 (9.4)	172 (13.8)
No biopsy record^e^	79 (38.9)	217 (33.6)	83 (21.0)	379 (30.5)

a The distribution of the PRS is based on the distribution in the underlying population of 24 617 genotyped men (6155 in the bottom, 12 308 in the middle, and 6154 in the top PRS quartile). IQR = interquartile range; mpMRI = multi-parametric magnetic resonance imaging; PI-RADS = Prostate Imaging–Reporting and Data System; PRS = polygenic risk score.

b Prostate-specific antigen was missing for 15 study participants.

c Of the 1243 men included, 1098 (88.3%) reported non-Hispanic, 5 (0.4%) Hispanic, and 140 (11.3%) had missing ethnicity.

d Other include the reported categories American Indian or Alaska Native, Native Hawaiian or Other Pacific Islander, Two or more, and Other (not reported separately due to few men in these categories).

e Among men with a PI-RADS 4-5 lesion, a biopsy record was available for 95.7% (94.4% of men in the bottom, 94.6% of men in the middle, and 97.8% of men in the top PRS quartile).

The PRS was calculated based on the multi-ancestry 400-variant prostate cancer PRS (6), of which 391 variants were available for analysis. As described in a previous publication (8) and in accordance with the original publication (6), a man’s PRS was constructed as the sum of risk allele dosages weighted by the log odds ratios for each variant. We categorized men according to PRS quartiles (bottom: 0%-25%, middle: 25%-75%, top: 75%-100%) and examined frequencies of mpMRI (based on the source population) and biopsy results (based on men who underwent mpMRI). Multivariable expanded data logistic regression (9) was used to estimate relative proportions and two-sided 95% confidence intervals (CI), with adjustment for age, self-reported race, and prostate-specific antigen level. Stratifications by race and calculations of the 451-variant PRS (including the additional 51 variants linked to prostate-specific antigen) were also conducted. Neither study participants nor physicians were aware of PRS-calculated genetic risk.

Men who underwent mpMRI had a median age of 66.0 years and a median prostate-specific antigen value of 5.8 ng/mL at the timepoint of the MRI (Table 1). In total, 69.5% of men had a biopsy. Compared with men in the bottom PRS quartile, men in the top PRS quartile were younger, had a higher prostate-specific antigen value, and an over-representation of Black men, in accordance with previous research showing a higher PRS among Black men (7). Both overall and when restricted to White men only, men in the top PRS quartile were more likely to have had an indication for and to undergo mpMRI (overall: 6.4% vs 3.3% in the bottom PRS quartile, adjusted relative proportion 1.99 [95% CI = 1.68 to 2.35]; White men: 6.6% vs 3.3% in the bottom PRS quartile, adjusted relative proportion 2.03 [95% CI = 1.70 to 2.42]) (Table 2 and Supplementary Table 1, available online).

**Table 2.** Number of men with an indication for and who underwent mpMRI and number of clinically significant prostate cancers together with corresponding relative proportions and 95% confidence intervals

	Bottom PRS (0%-25%)	Middle PRS (25%-75%)	Top PRS (75%-100%)
Indication for and underwent mpMRI			
No. (%)	203/6155 (3.3)	645/12308 (5.2)	395/6154 (6.4)
Relative proportion (95% CI)^a^	1 (Ref.)	1.59 (1.36 to 1.86)	1.99 (1.68 to 2.35)
Clinically significant cancer			
No. (%)	58/203 (28.6)	228/645 (35.3)	186/395 (47.1)
Relative proportion (95% CI)^b^	1 (Ref.)	1.21 (0.96 to 1.54)	1.72 (1.35 to 2.19)
Clinically significant cancer by PI-RADS score			
No. (%)			
≤2	4/93 (4.3)	24/290 (8.3)	15/135 (11.1)
3	0/21 (0)	10/61 (16.4)	9/33 (27.3)
4-5	54/89 (60.7)	194/294 (66.0)	162/227 (71.4)
Clinically significant cancer for PI-RADS 3-5 combined			
No. (%)	54/110 (49.1)	204/355 (57.5)	171/260 (65.8)
Relative proportion (95% CI)^b^	1 (Ref.)	1.14 (0.93 to 1.40)	1.38 (1.12 to 1.69)

a Adjusted for age and race. CI = confidence interval; mpMRI = multi-parametric magnetic resonance imaging; PI-RADS = Prostate Imaging–Reporting and Data System; PRS = polygenic risk score.

b Adjusted for age, prostate-specific antigen level, and race.

Among all men who underwent mpMRI, men in the top PRS quartile were more likely to have clinically significant prostate cancer (47.1% vs 28.6% in the bottom PRS quartile, adjusted relative proportion 1.72 [95% CI = 1.35 to 2.19]) (Table 2). Among men with a positive mpMRI (PI-RADS 3-5), men in the top PRS quartile had the highest frequency of clinically significant prostate cancer (65.8% vs 49.1%, adjusted relative proportion 1.38 [95% CI = 1.12 to 1.69]). (PI-RADS score 3 was originally intended to be examined separately for this calculation but was due to sparsity of data combined with PI-RADS 4-5 in accordance with work by others (10)). Among men with a negative mpMRI (PI-RADS ≤2), the same pattern was observed, with men in the top PRS quartile having the highest frequency of clinically significant cancer (11.1% vs 4.3%).

Although the numbers only allowed for calculations of adjusted relative proportions for White men, percentages were similar in the other racial groups, in particular where the numbers were highest (Supplementary Table 2, available online). For example, among who underwent mpMRI in the top PRS quartile, 41.2% of Black men and 47.7% of White men had clinically significant cancer.

To examine how the PRS could contribute toward reducing the number of clinically significant cancers missed by mpMRI, we constructed a PRS and mpMRI-based scenario for selecting men to undergo biopsy (Supplementary Table 3, available online). If all men in the top PRS group were to undergo a biopsy, and men with PI-RADS 3-5 in the other PRS groups, the overall number of missed clinically significant cancers would be reduced from 9.1% to 5.9%, but at the cost of increasing the number of low-grade and negative biopsies from 40.8% to 48.4%. The latter was reduced to 47.1% if men in the bottom PRS group with a PI-RADS 3 were not selected for biopsy.

Compared with the 400-variant PRS, the 451-variant PRS demonstrated a stronger association for having an indication for and to undergo mpMRI, but a weaker association for clinically significant cancer (Supplementary Table 4, available online). In the scenario described above, test characteristics were marginally in favor for the 400-variant PRS: the number of missed clinically significant cancers were the same (5.9%), but the number of low-grade and negative biopsies performed increased to 50.0% with the 451-variant PRS (Supplementary Table 3, available online).

Our study shows that among men selected for and who underwent mpMRI, men with a high PRS were more likely to have clinically significant prostate cancer than men with a low PRS. Our study further indicates that men with a high PRS are more likely to have clinically significant cancer missed by mpMRI. However, to avoid overdiagnosis of low-grade cancers, future work should focus developing PRS that are more specific for aggressive prostate cancer. Although the optimal use of the PRS is yet to be determined in prospective studies, the PRS may have several potential clinical applications, including providing guidance in determining which men to target for biopsy and repeat mpMRI, and may, if our results are replicated, be relevant in the management of PI-RADS 3, for which there are no consensus guidelines. Although no firm conclusions can be drawn from our study due to the limited sample size for other groups than White men, the multi-ancestry PRS may potentially be a universal clinical tool that can be applied across populations.

One application is the integration into screening for prostate cancer, where men with a high PRS undergo and men with a low PRS and no other indication are spared further testing. Although the interest in integrating a PRS into diagnostic procedures and screening for prostate cancer is growing, as demonstrated by ongoing prospective evaluations (11-13), few studies have been published to date. The Stockholm 3 (STHLM3 and STHLM3-MRI) trials showed that the Stockholm3 test (which includes a smaller subset of genetic risk variants but also other markers) can improve detection of clinically significant prostate cancer compared with prostate-specific antigen alone (14,15).

Our study has limitations. This study was not designed for evaluating a screening strategy of a PRS combined with mpMRI and included a selected group of men who may not be representative of other men with an indication for mpMRI. Compared with the overall pool of Mass General Brigham patients, individuals included in the biobank have been found to have a higher health-care utilization and a higher disease burden (16). This may have resulted in overestimated detection rates for clinically significant prostate cancer in our analysis, although previous findings based on the PRS have generally been consistent across studies (including from the Mass General Brigham Biobank), with slight variation in magnitude but not direction (6,8). Nevertheless, our study is the first to examine the 400-variant PRS in a clinical setting with a sample size of imaged individuals comparable to those reported for large population-based screening trials (10,14).

In conclusion, our study provides initial support for using the multi-ancestry PRS to improve identification of clinically significant prostate cancer. Prospective studies are needed to confirm this finding, and to help elucidate whether improved detection of aggressive cancer will translate to better survival outcomes.

## Supplementary Material

pkae014_Supplementary_Data

## Data Availability

Data can be obtained from the Mass General Brigham Biobank (https://personalizedmedicine.partners.org/biobank/), but restrictions apply to the availability of these data and data are not publicly available. Data are available for all Mass General Brigham investigators and their external affiliates, including academic and commercial affiliates, provided a protocol from the Institutional Review Board (IRB) and a data use agreement. Investigators can contact biobank@partners.org for more information.
