# Supplementary material for: Combining magnetic resonance imaging with a multi-ancestry polygenic risk score to improve identification of clinically significant prostate cancer
Source: JNCI Cancer Spectr. 2024 Mar 1;8(2):pkae014. doi: 10.1093/jncics/pkae014 (PMC10980589; doi:10.1093/jncics/pkae014)
Supplement: pkae014_Supplementary_Data [file pkae014_supplementary_data.pdf]

Supplementary Methods

Data extraction

PI-RADS scores were extracted from diagnostic prostate mpMRIs using CEBI-Miner, a natural language processing tool developed at Brigham and Women Hospital's Center for Evidence-Based Imaging. The highest PI-RADS score in each report was obtained and in case of multiple reports, the report with the highest PI-RADS score was kept.

Biopsy information and Gleason scores were extracted from electronic health records and pathology reports, filtered on reports +/- 1 year from the date of the mpMRI. In case of multiple biopsies, the biopsy in closest proximity to the mpMRI was used, at a median time of 0.9 months post-mpMRI. Biopsy results including a grade group of 1 or higher were classified as positive (with grade group 2 or higher defined as clinically significant prostate cancer) and biopsy results only reporting benign lesions as negative. Men with no documented biopsy were classified as having no biopsy. Reports of PI-RADS 3-5 scores classified as negative or no biopsy were manually validated against patient charts.

Prostate-specific antigen values were extracted from electronic health records (measured at a median time of 1.0 months pre-mpMRI).

Supplementary Table 1. Results for the 400-variant PRS for White men.

	Bottom PRS (0%-25%)	Middle PRS (25%-75%)	Top PRS (75%-100%)
Indication for and underwent mpMRI			
No. (%)	185/5650 (3.3)	599/11080 (5.4)	342/5151 (6.6)
Relative proportion (95% CI) [a]	1 (Ref.)	1.65 (1.40 to 1.94)	2.03 (1.70 to 2.42)
Clinically significant cancer			
No. (%)	57/185 (30.8)	216/599 (36.1)	163/342 (47.7)
Relative proportion (95% CI) [b]	1 (Ref.)	1.16 (0.91 to 1.48)	1.61 (1.26 to 2.05)
Clinically significant cancer by PI-RADS score			
No. (%)			
≤2	4/86 (4.7)	23/267 (8.6)	13/114 (11.4)
3	0/15 (0)	10/58 (17.2)	8/28 (28.6)
4-5	53/84 (63.1)	183/274 (66.8)	142/200 (71.0)
Clinically significant cancer for PI-RADS 3-5 combined			
No. (%)	53/99 (53.5)	193/332 (58.1)	150/228 (65.8)
Relative proportion (95% CI) [b]	1 (Ref.)	1.13 (0.92 to 1.39)	1.32 (1.07 to 1.63)

Abbreviations: CI, confidence interval; mpMRI, multi-parametric magnetic resonance imaging; PI-RADS, prostate imaging–reporting and data system; PRS, polygenic risk score.

[a] Adjusted for age.

[b] Adjusted for age and prostate-specific antigen level.

Supplementary Table 2. Descriptive results for the 400-variant PRS by racial groups.

	Bottom PRS (0%-25%)	Middle PRS (25%-75%)	Top PRS (75%-100%)
Indication for and underwent mpMRI, n (%)			
Asian	6/140 (4.3)	8/225 (3.6)	3/66 (4.5)
Black	1/42 (2.4)	8/294 (2.7)	34/570 (6.0)
White	185/5650 (3.3)	599/11080 (5.4)	342/5151 (6.6)
Other	3/163 (1.8)	12/346 (3.5)	11/206 (5.3)
Missing	8/160 (5.0)	18/363 (5.0)	5/161 (3.1)
Clinically significant cancer, n (%)			
Asian	0/6 (0)	1/8 (12.5)	2/3 (66.7)
Black	1/1 (100)	2/8 (25.0)	14/34 (41.2)
White	57/185 (30.8)	216/599 (36.1)	163/342 (47.7)
Other	0/3 (0)	3/12 (25.0)	5/11 (45.5)
Missing	0/8 (0)	6/18 (33.3)	2/5 (40.0)
Clinically significant cancer by PI-RADS score, n (%)			
Asian			
≤2	0/2 (0)	0/5 (0)	0/0 (0)
3	0/1 (0)	0/1 (0)	1/2 (50.0)
4-5	0/3 (0)	1/2 (50.0)	1/1 (100)
Black			
≤2	0/0 (0)	0/2 (0)	2/17 (11.8)
3	0/0 (0)	0/2 (0)	0/1 (0)
4-5	1/1 (100)	2/4 (50.0)	12/16 (75.0)
White			
≤2	4/86 (4.7)	23/267 (8.6)	13/114 (11.4)
3	0/15 (0)	10/58 (17.2)	8/28 (28.6)
4-5	53/84 (63.1)	183/274 (66.8)	142/200 (71.0)
Other			
≤2	0/2 (0)	1/7 (14.3)	0/3 (0)
3	0/1 (0)	0/0 (0)	0/2 (0)
4-5	0/0 (0)	2/5 (40.0)	5/6 (83.3)
Missing			
≤2	0/3 (0)	0/9 (0)	0/1 (0)
3	0/4 (0)	0/0 (0)	0/0 (0)
4-5	0/1 (0)	6/9 (66.7)	2/4 (50.0)

Abbreviations: mpMRI, multi-parametric magnetic resonance imaging; PI-RADS, prostate imaging–reporting and data system; PRS, polygenic risk score.

Supplementary Table 3. Evaluation of potential strategies for selecting men to undergo biopsy. The calculation is based on the numbers presented in Table 2 and Supplementary Table 4, under the assumption that all clinically significant cancers have been identified.

Strategy for selecting men to undergo biopsy	Missed clinically significant cancers	Sensitivity	Positive predictive value	Performed grade group 1 or negative biopsies	Specificity	Negative predictive value
All men: PI-RADS 3-5	9.1%	90.9%	59.2%	40.8%	61.6%	91.7%
400-variant PRS-based strategy:						
Bottom PRS: PI-RADS 3-5<br>Middle PRS: PI-RADS 3-5<br>Top PRS: all men	5.9%	94.1%	51.6%	48.4%	46.0%	92.7%
Bottom PRS: PI-RADS 4-5<br>Middle PRS: PI-RADS 3-5<br>Top PRS: all men	5.9%	94.1%	52.9%	47.1%	48.8%	93.1%
451-variant PRS-based strategy:						
Bottom PRS: PI-RADS 3-5<br>Middle PRS: PI-RADS 3-5<br>Top PRS: all men	5.9%	94.1%	50.0%	50.0%	42.4%	92.1%
Bottom PRS: PI-RADS 4-5<br>Middle PRS: PI-RADS 3-5<br>Top PRS: all men	6.1%	93.9%	51.0%	49.0%	44.9%	92.3%

Abbreviations: PI-RADS, prostate imaging–reporting and data system; PRS, polygenic risk score.

Supplementary Table 4. Results for the 451-variant PRS (all men).

	Bottom PRS (0%-25%)	Middle PRS (25%-75%)	Top PRS (75%-100%)
Indication for and underwent mpMRI			
No. (%)	176/6155 (2.9)	640/12308 (5.2)	427/6154 (6.9)
Relative proportion (95% CI) [a]	1 (Ref.)	1.83 (1.55 to 2.16)	2.54 (2.14 to 3.02)
Clinically significant cancer			
No. (%)	54/176 (30.7)	238/640 (37.2)	180/427 (42.2)
Relative proportion (95% CI) [b]	1 (Ref.)	1.26 (0.99 to 1.60)	1.47 (1.15 to 1.89)
Clinically significant cancer, by PI-RADS score			
No. (%)			
≤2	4/80 (5.0)	24/275 (8.7)	15/163 (9.2)
3	1/20 (5.0)	8/59 (13.6)	10/36 (27.8)
4-5	49/76 (64.5)	206/306 (67.3)	155/228 (68.0)
Clinically significant cancer for PI-RADS 3-5 combined			
No. (%)	50/96 (52.1)	214/365 (58.6)	165/264 (62.5)
Relative proportion (95% CI) [b]	1 (Ref.)	1.16 (0.94 to 1.42)	1.27 (1.02 to 1.56)

Abbreviations: CI, confidence interval; mpMRI, multi-parametric magnetic resonance imaging; PI-RADS, prostate imaging–reporting and data system; PRS, polygenic risk score.

[a] Adjusted for age and race.

[b] Adjusted for age, prostate-specific antigen level, and race.
